# Transactions of the State Medical Society

**Published:** 1856-07

**Authors:** 


ART. IV.— *Transactions of the State Medical Society.* Albany: C. VAN BENTHUYSEN, Printer to the Legislature.

The temptation to abuse this volume is a little too strong for our continence. To see such men as the President, Prof. Hamilton, Profs. Coventry and McNaughton, Dr. Blatchford and others immortalized in whitey-brown paper, battered type, and infamous proof-reading, is rather more than we can submit to pleasantly. Mr. C. Van Benthuysen must have an immense regard for his typographical reputation when he sends out such a thing as this to represent a venerable and learned society. Hereafter if the assistance of the Legislature does not enable the society to get out a reputable volume of transactions, the society had better find some other means of publishing. Assess the county societies, make contributors pay the printer, do anything rather than submit to such typography as this.

The society of Southern Central New York manage some how, without outside assistance, to publish, yearly, a volume as large as this, in very handsome style. We would suggest that Drs. Orton, of Binghampton, and Hyde and Green, of Cortland, be appointed a committee on the part of the state society, to inform it how the thing is done.

We are duly thankful that the literary merits of the transactions are not to be judged by its appearance. The eulogium on the life and services of T. Romeyn Beck, by the President, Prof. Hamilton, has the distinguishing merit of being a plain, unvarnished tale, telling us what Dr. Beck had done.

The eulogist seems forgetful of himself, and so absorbed in his subject as to neglect all oratorical display which might serve to call attention from the man to the speaker. Those familiar with Prof. Hamilton's capacity as an orator will (except in the beautiful exordium) fail to recognize the usual fire of his eloquence.

Prof. Coventry follows with an excellent paper on tuberculosis. It is pleasant to notice the hopeful tone in which our most careful and competent writers now speak of consumption. No longer considered as hopeless or intractable, we find the most sober-minded men—like Prof. Coventry—hoping much and toiling much for its cure. We commend the leading idea of Prof. C.'s treatment to the earnest consideration of our readers. It is exercise and food—in a word, hygiene. In speaking of pure tuberculosis he says: "No medicine is required, unless there is derangement of the digestive organs, which should, if possible, be corrected; the patient must depend on hygienic means, and not on medicine, for his recovery."

The "Report on Rest and the Abolition of Pain in the Treatment of Disease," by Dr. Thomas W. Blatchford, of Troy, is also deserving of warm commendation. Aside from the mere fact that the relief of pain is one of the greatest duties of the physician, Dr. Blatchford is an earnest advocate of the doctrine that rest and quiet are essential to the *cure* of disease. The *suaviter in modo* is rapidly replacing the rough, vigorous, heroic treatment of disease: a fact for which to be grateful for, however brilliant *occasional results* may be obtained from active interference with powerful perturbants, a far more desirable *general result* may be reached by gentler means.

Following Dr. Blatchford's report, Dr. A. L. Saunders sends a brief apology for withholding a report on pneumonia. Dr. Saunders states that he has nothing to offer different from the "principles so well set forth by the late lamented Dr. Swett," to whose work he refers the society, "and also to an able report of a committee of the Buffalo Medical Association, published in the October number of the Buffalo Medical Journal for 1855." In this reference Dr. Saunders endorses the non-sanguinary treatment of pneumonia, even as it is found among the hills of Madison county. We hear so much of the distinction between pneumonia in city and in country, that we should have been glad to see Dr. Saunders' views fully presented. However, we must rest on our own experience of seven years among the hills, during which we found no greater use for the lancet than in our later practice in hospital or city alley.

Prof. Howard Townsend contributes a well-written paper on Malignant Pustule, after which we have a "document" by Dr. Thomas Goodsell, denominated "Fœtation, from Coition to Parturition. We protest against such a good-natured committee of publication. There should be some censorship over papers in these transactions. There is nothing in Dr. Goodsell's "report" which is either new or interesting, and we should be sorry to see it received as an index of the physiological knowledge of the profession of New York.

Prof. Alden March (what would a volume of transactions be without a paper from him?) sends in an interesting case of encysted osseous tumor, which will repay perusal.

Dr. Phelps has a "Historic notice of Dislocations of the Femur," intended to deprive Dr. Reid, of Rochester, of any honor in connection with the new method of reduction. Where Dr. Reid learned his method we have no means of knowing, but it is a perfect marvel that so many old gentlemen should have known all about it for the last half century, and never have thought to mention it till since Dr. Reid's publication. A method, new at least to all practical surgeons, is published; when presto! up start a dozen bald-headed old gentlemen, with a chorus of "Bless you! we knew all about that when we were students!" It's a pity they forgot it so easily, or failed to make any use of their precious information.

One or two other papers of little general interest complete the volume. It may be judged from our cursory notice that, in our opinion, the proceedings this year are

> "like Jeremiah's figs;
> The good are very good indeed,
> The bad, too bad for pigs!"

